# 中西方骨髓增生异常肿瘤临床和实验室特征及生存的比较研究

**DOI:** 10.3760/cma.j.cn121090-20241210-00555

**Published:** 2025-03

**Authors:** 琳琳 刘, 冰 李, 铁军 秦, 泽锋 徐, 士强 曲, 丽娟 潘, 清妍 高, 蒙 焦, 玉娇 贾, 承文 李, 琦 孙, 慧君 王, 志坚 肖

**Affiliations:** 1 中国医学科学院血液病医院（中国医学科学院血液学研究所），血液与健康全国重点实验室，国家血液系统疾病临床医学研究中心，细胞生态海河实验室，天津 300020 State Key Laboratory of Experimental Hematology, National Clinical Research Center for Blood Diseases, Haihe Laboratory of Cell Ecosystem, Institute of Hematology & Blood Diseases Hospital, Chinese Academy of Medical Sciences & Peking Union Medical College, Tianjin 300020, China; 2 天津医学健康研究院，天津 301600 Tianjin Institutes of Health Science, Tianjin 301600, China

**Keywords:** 骨髓增生异常肿瘤, 细胞遗传学, 基因突变, 预后, Myelodysplastic neoplasm, Cytogenetics, Mutational profile, Prognosis

## Abstract

**目的:**

比较中西方骨髓增生异常肿瘤（MDS）临床和实验室特征及生存特点。

**方法:**

纳入自2016年8月至2024年6月于中国医学科学院血液病医院确诊的1 464例原发性初治成人MDS患者和国际预后工作组（IWG-PM）的2 191例MDS患者，回顾性分析比较两组患者临床和实验室特征及生存情况。

**结果:**

我中心患者较IWG-PM患者更年轻（中位年龄56岁对72岁，*P*<0.001）；三系血细胞计数更低（均*P*<0.001）；染色体核型出现单独del（20q）、+8和复杂核型的比例较高，正常核型、del（5q）、−Y比例较低（均*P*<0.001）；U2AF1、NRAS、NPM1基因突变率较高（均*P*<0.05），而ASXL1、SF3B1、RUNX1等基因突变率较低（*P*值均<0.05）；总生存（OS）期与IWG-PM组患者相比差异无统计学意义［48（95％ *CI* 40～56）个月对45（95％ *CI* 40～49）个月，*P*＝0.449］。年龄≤45岁的患者中，我中心患者有更高比例的染色体+8（*P*＝0.070）和U2AF1基因突变（*P*<0.001），4年OS率更高（75.5％对62.1％，*P*＝0.001）；年龄≥70岁的患者中，我中心患者del（20q）和复杂核型检出率更高（*P*值均<0.05），del（5q）和正常核型检出率更低（*P*值均<0.05），NPM1基因突变率较高（*P*＝0.004），SF3B1和TET2突变率较低（*P*值均<0.05），OS期更短［20（95％ *CI* 13～27）个月对37（95％ *CI* 32～42）个月，*P*<0.001］。

**结论:**

我国与西方MDS患者在发病年龄、临床特征及细胞分子遗传学异常等实验室特征方面存在差异，年龄相近的中西方患者间差异仍然显著。中西方患者总体生存无显著差异，但年轻和老年患者的生存期在中西方之间存在差异。

## Abstract (en)

**Objective:**

To compare the clinical and laboratory characteristics and survival between Chinese and Western patients with myelodysplastic neoplasms（MDS）.

**Methods:**

Clinical and laboratory data were collected from 1,464 primary adult patients diagnosed with MDS at the Institute of Hematology & Blood Diseases Hospital from August 2016 to June 2024. Collected data were retrospectively analyzed and compared with 2,191 patients from the International Working Group for the Prognosis of Myelodysplastic Syndromes（IWG-PM）.

**Results:**

Chinese patients were significantly younger（median age: 56 years *vs*. 72 years, *P*<0.001）and experienced more severe hematopenia（*P*<0.001）compared with patients from the IWG-PM. Further, Chinese patients exhibited a higher percentage of isolated del（20q）, +8, and complex karyotypes as well as a lower percentage of normal karyotypes, del（5q）, and −Y（*P*<0.001）. Higher U2AF1, NRAS, and NPM1 mutation rates and lower ASXL1, SF3B1, and RUNX1 mutation rates were observed in Chinese patients than in participants from the IWG-PM（*P*<0.05）. No significant difference in overall survival（OS）was found between the two groups（median OS: 48［95％ *CI*: 40–56］months, *vs*. 45［95％ *CI*: 40–49］months; *P*＝0.449）. Among participants aged ≤45 years, Chinese patients demonstrated more trisomy 8（*P*＝0.070）and U2AF1 mutation（*P*<0.001）and higher 4-year OS rate compared with those from the IWG-PM（75.5％ *vs*. 62.1％, *P*＝0.001）. Among participants aged ≥70 years, Chinese patients exhibited more complex karyotypes but fewer del（5q）as well as more NPM1 but less SF3B1 and TET2 compared with those from the IWG-PM（*P*<0.05）. Chinese patients demonstrated shorter survival（median OS: 20［95％ *CI*: 13–27］months *vs*. 37［95％ *CI*: 32–42］months, *P*<0.001）.

**Conclusion:**

Chinese and Western MDS patients differ in age of onset, clinical features, and cytogenetic or molecular genetic abnormalities, with significant differences persisting in age-matched groups. Although the OS is similar, disparities exist in survival for younger and older patients between the two populations.

骨髓增生异常肿瘤（Myelodysplastic neoplasm, MDS）[1]，亦称骨髓增生异常综合征（Myelodysplastic syndromes, MDS），是一组高度异质性的克隆性血液系统肿瘤，以血细胞减少、发育异常和向骨髓衰竭或急性髓系白血病转化为特征[2]。MDS发病以老年人为主，且发病率随年龄增长而增加[3]，西方国家如美国、德国等较大队列研究显示MDS中位诊断年龄为70岁左右[4]–[7]，但我国研究队列报道的MDS患者普遍更年轻，且临床表现及实验室特征与国外患者不完全相同[8]–[11]。目前国内尚无大队列研究系统比较中国与西方国家MDS患者临床、实验室特征及生存情况。因此，本研究回顾性对比分析在本中心诊治的MDS患者与MDS国际预后工作组（International Working Group for Prognosis in MDS, IWG-PM）队列，探讨中西方MDS的异同。

## 病例与方法

一、病例资料

研究纳入自2016年8月至2024年6月于中国医学科学院血液病医院MDS和MPN诊疗中心资料完整的1 464例原发性初治成人MDS患者，所有患者均按照WHO 2016标准[12]进行分型诊断。西方国家MDS患者选自IWG-PM队列，根据参考文献[5]自网站（https://www.cbioportal.org/study/summary?id＝mds_iwg_2022）筛选出2 191例原发MDS患者，获取其临床及实验室检查数据。

二、染色体核型分析

应用短期培养法常规制备染色体标本，R显带法分析核型，核型描述符合人类细胞遗传学国际命名体制（ISCN2016）[13]。按照修订的MDS国际预后积分系统（IPSS-R）[7]对染色体核型进行预后分组。

三、基因突变二代测序

分离患者骨髓单个核细胞提取基因组DNA，针对141个或267个血液肿瘤相关基因编码序列进行测序，利用CCDS、dbSNP（v138）、COSMIC等数据库进行生物信息学分析。具体方法见本中心此前已发表文献[9]。

四、预后评估

本中心共有1 345例（91.9％）患者有可供评估的实验室及染色体核型结果，按照IPSS-R进行评分和预后分组；IPSS-R评分≤3.5分为较低危组，评分>3.5分为较高危组；共有1 309例（89.4％）完善二代测序检测的患者同时采用分子学国际预后积分系统（IPSS-M）[5]进行预后分组，IPSS-M评分≤0分为较低危组，评分>0分为较高危组。

五、治疗

本中心共1 326例（90.6％）患者可随访到治疗方案。其中69例（5.2％）患者等待与观察，168例（12.7％）接受促造血±输血治疗，385例（29.1％）行免疫调节/抑制剂±促造血治疗，283例（21.3％）行地西他滨或阿扎胞苷±维奈克拉去甲基化治疗，56例（4.2％）接受联合化疗，281例（21.2％）接受移植，63例（4.8％）服中药治疗，21例（1.4％）入组临床试验。

六、随访

随访截止时间为2024年10月25日，中位随访时间为26（*IQR* 23.9～28.1）个月，共70例（4.7％）患者失访。随访资料来源于住院和门诊病历及电话随访记录。总体生存时间（Overall survival, OS）定义为自诊断日期到死亡或造血干细胞移植或末次随访日期。

七、统计学处理

不符合正态分布的连续变量的以*M*（*IQR*）描述，通过Mann-Whitney *U*检验进行组间比较；分类变量以频数和百分比（％）描述，通过Fisher确切概率法或蒙特卡洛渐进法进行比较。采用Kaplan-Meier法绘制生存曲线，双侧*P*<0.05认为差异有统计学意义。使用SPSS 26.0进行统计分析，应用Graphpad Prism 8.0进行绘图。

## 结果

一、本中心与IWG-PM队列整体对比

1. 临床特征：如表1所示，本中心1 464例原发MDS患者相较2 191例IWG-PM患者更年轻（中位年龄56岁对72岁，*P*<0.001），我中心≤45岁年轻患者占25.8％，≥70岁老年患者占11.5％；而IWG-PM中≤45岁患者占5.0％，≥75岁患者占57.5％。本中心患者三系血细胞计数更低（*P*值均<0.001），应用WHO 2016诊断分型，我中心相较IWG-PM有更多患者诊断为MDS伴多系血细胞发育异常（MDS-MLD）、MDS伴原始细胞增多（MDS-EB），更少诊断为MDS伴环状铁粒幼红细胞（MDS-RS-SLD/MLD）和MDS伴单纯del（5q）［MDS-del（5q）］（*P*<0.001）。

**表1:** 本中心MDS患者与IWG-PM患者临床特征比较

临床特征	本中心患者（1 464例）	IWG-PM患者（2 191例）	*P*值
性别［例（％）］			0.153
男性	935（63.9）	1 347（61.5）	
女性	529（36.1）	844（38.5）	
年龄［岁，*M*（*IQR*）］	56（45, 64）	72（64, 79）	<0.001
≤45岁［例（％）］	377（25.8）	110（5.0）	<0.001
≥70岁［例（％）］	168（11.5）	1 259（57.5）	<0.001
外周血细胞计数			
WBC［×10^9^/L，*M*（*IQR*）］	2.61（1.82, 3.74）	3.78（2.60, 5.57）	<0.001
ANC［×10^9^/L，*M*（*IQR*）］	1.10（0.63, 2.02）	1.84（1.00, 3.02）	<0.001
HGB［g/L，*M*（*IQR*）］	79.0（66.0, 97.0）	96（84.8, 110.0）	<0.001
PLT［×10^9^/L，*M*（*IQR*）］	64（34, 130）	128（68, 227）	<0.001
骨髓原始细胞比例［％，*M*（*IQR*）］	2.5（1.0, 7.0）	3.0（1.0, 7.0）	0.002
WHO 2016诊断分型［例（％）］			<0.001
MDS-SLD	126（8.6）	202（9.2）	
MDS-RS-SLD	52（3.6）	233（10.6）	
MDS-MLD	517（35.3）	582（26.6）	
MDS-RS-MLD	85（5.8）	198（9.0）	
MDS-EB1	301（20.6）	386（17.6）	
MDS-EB2	309（21.1）	387（17.7）	
MDS-del（5q）	23（1.6）	126（5.8）	
MDS-U	51（3.5）	77（3.5）	

**注** MDS：骨髓增生异常肿瘤；IWG-PM：MDS国际预后工作组队列；MDS-SLD：MDS伴单系血细胞发育异常；MDS-RS-SLD：MDS伴环状铁粒幼红细胞伴单系血细胞发育异常；MDS-MLD：MDS伴多系血细胞发育异常；MDS-RS-MLD：MDS伴环状铁粒幼红细胞伴多系血细胞发育异常；MDS-EB1：MDS伴原始细胞增多1型；MDS-EB2：MDS伴原始细胞增多2型；MDS-del（5q）：MDS伴单纯del（5q）；MDS-U：MDS不能分类型

2. 细胞遗传学特征：染色体核型结果显示，对于单独出现的染色体核型异常，我中心患者相较IWG-PM患者有更高比例的del（20q）（5.8％对2.4％，*P*<0.001）、+8（9.6％对4.2％，*P*<0.001）和复杂核型（15.4％对9.3％，*P*<0.001），更低比例的正常核型（49.1％对61.2％，*P*<0.001）、del（5q）（2.4％对7.4％，*P*<0.001）和−Y（1.3％对4.0％，*P*<0.001），−7/del（7q）比例两组间差异无统计学意义（表2）。按照IPSS-R进行预后分组，IWG-PM组较低危组患者比例更高（60.4％对42.1％，*P*<0.001）。

**表2:** 本中心MDS患者与IWG-PM患者细胞遗传学特征及IPSS-R、IPSS-M分级比较

指标	总体患者			≤45岁患者			≥70岁患者		
	本中心（1 345例）	IWG-PM（2 113例）	*P*值	本中心（345例）	IWG-PM（108例）	*P*值	本中心（149例）	IWG-PM（1 215例）	*P*值
染色体核型［例（％）］									
正常核型	661（49.1）	1 293（61.2）	<0.001	159（46.8）	63（58.3）	0.046	73（50.0）	739（60.8）	0.013
−Y	17（1.3）	84（4.0）	<0.001	3（0.9）	1（0.9）	1.000	6（4.1）	60（4.9）	0.839
del（5q）	32（2.4）	156（7.4）	<0.001	8（2.4）	3（2.8）	0.731	2（1.4）	98（8.1）	0.001
del（20q）	78（5.8）	51（2.4）	<0.001	19（5.6）	1（0.9）	0.057	11（7.5）	40（3.3）	0.016
+8	129（9.6）	89（4.2）	<0.001	40（11.8）	6（5.6）	0.070	9（6.2）	54（4.4）	0.401
−7/del（7q）	28（2.1）	43（2.0）	1.000	7（2.1）	3（2.8）	0.710	3（2.1）	18（1.5）	0.486
复杂核型	207（15.4）	196（9.3）	<0.001	41（12.1）	13（12.0）	1.000	29（19.9）	108（8.9）	<0.001
细胞遗传学分组［例（％）］			<0.001			0.252			<0.001
极好	19（1.4）	94（4.4）		4（1.2）	1（0.9）		6（4.1）	65（5.3）	
好	769（57.2）	1503（71.1）		187（54.2）	68（63.0）		87（58.4）	873（71.9）	
中等	324（24.1）	269（12.7）		100（29.0）	21（19.4）		25（16.8）	148（12.2）	
差	89（6.6）	95（4.5）		34（9.9）	9（8.3）		4（2.7）	44（3.6）	
极差	144（10.7）	152（7.2）		20（5.8）	9（8.3）		27（18.1）	85（7.0）	
IPSS-R预后分组［阳性例数/总例数（％）］			<0.001			0.101			<0.001
极低危	58/1 341（4.3）	344/2 090（16.5）		18/344（5.2）	11/103（10.7）		6/149（4.0）	215/1 206（17.8）	
低危	351/1 341（26.2）	825/2 090（39.5）		90/344（26.2）	34/103（33.0）		39/149（26.2）	508/1 206（42.1）	
中危	405/1 341（30.2）	442/2 090（21.1）		108/344（31.4）	21/103（20.4）		36/149（24.2）	237/1 206（19.7）	
高危	294/1 341（21.9）	287/2 090（13.3）		77/344（22.4）	24/103（23.3）		35/149（23.5）	136/1 206（11.3）	
极高危	233/1 341（17.4）	201/2 090（9.6）		51/344（14.8）	13/103（12.6）		33/149（22.1）	110/1 206（9.1）	
IPSS-M预后分组［阳性例数/总例数（％）］			<0.001			0.014			<0.001
极低危	35/1 309（2.7）	260/2 033（12.8）		12/337（3.6）	11/102（10.8）		1/146（0.7）	145/1 180（12.3）	
低危	247/1 309（18.9）	672/2 033（33.1）		64/337（19.0）	28/102（27.5）		27/146（18.5）	403/1 180（34.2）	
中低危	199/1 309（15.2）	244/2 033（12.0）		51/337（15.1）	9/102（8.8）		17/146（11.6）	147/1 180（12.5）	
中高危	195/1 309（14.9）	219/2 033（10.8）		62/337（18.4）	13/102（12.7）		22/146（15.1）	115/1 180（9.7）	
高危	315/1 309（24.1）	285/2 033（14.0）		77/337（22.8）	17/102（16.7）		31/146（21.2）	170/1 180（14.4）	
极高危	318/1 309（24.3）	353/2 033（17.4）		71/337（21.1）	24/102（23.5）		48/146（32.9）	200/1 180（16.9）	

**注** MDS：骨髓增生异常肿瘤；IWG-PM：MDS国际预后工作组队列；IPSS-R：修订的MDS国际预后积分系统；IPSS-M：分子学国际预后积分系统

3. 分子遗传学特征：基因突变二代测序结果显示，我中心MDS患者最常发生的基因突变有U2AF1（22.1％）、ASXL1（20.7％）、SF3B1（11.3％）、RUNX1（10.3％）和TET2（9.8％）。与IWG-PM患者相比，我中心患者有更高比例的U2AF1（22.1％对9.2％，*P*<0.001）、NRAS（4.6％对3.1％，*P*＝0.031）和NPM1（3.5％对1.3％，*P*<0.001）突变，而ASXL1（20.7％对26.4％，*P*<0.001）、SF3B1（11.3％对26.0％，*P*<0.001）、RUNX1（10.3％对12.6％，*P*＝0.040）、TET2（9.8％对29.0％，*P*<0.001）、DNMT3A（8.7％对17.2％，*P*<0.001）、SRSF2（5.4％对15.0％，*P*<0.001）等基因突变率更低（图1A）。两组患者IPSS-M预后分组差异有统计学意义（*P*<0.001）（表2），IWG-PM组较低危组患者比例更高（57.8％对36.7％，*P*<0.001）。

**图1:**
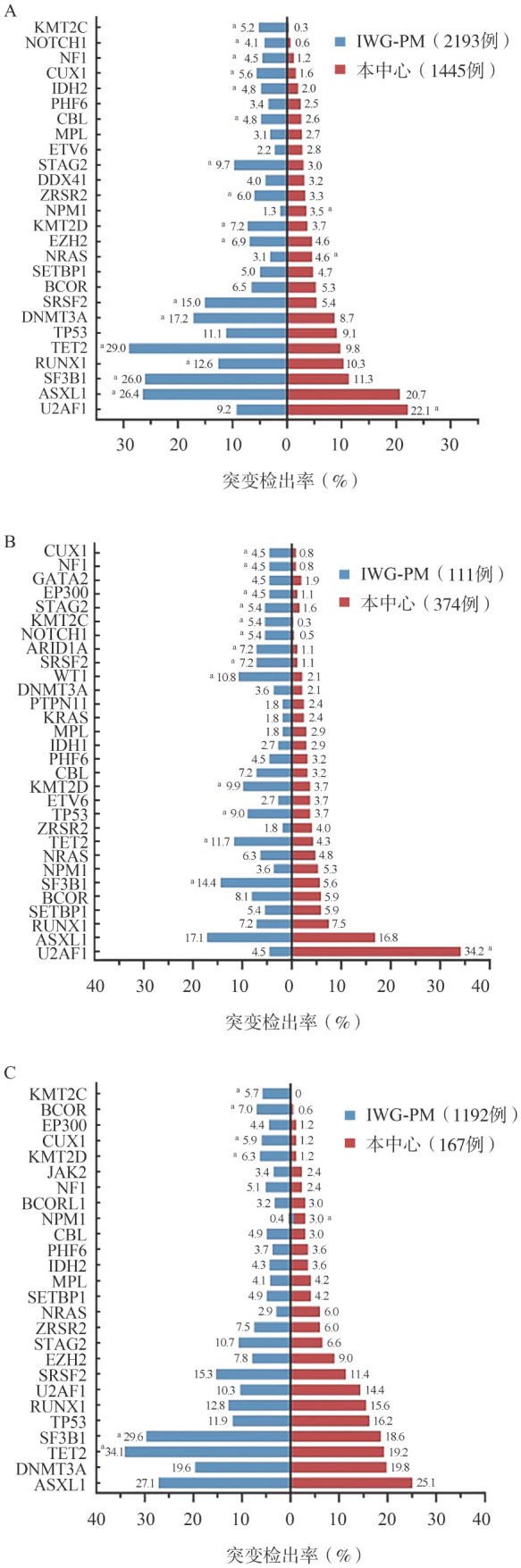
本中心MDS患者与IWG-PM患者基因突变情况对比（^a^*P*<0.05） **A** 总体患者；**B** 年龄≤45岁亚组；**C** 年龄≥70岁亚组 **注** MDS：骨髓增生异常肿瘤；IWG-PM：MDS国际预后工作组队列

4. 生存分析：我中心1 394例患者和IWG-PM组2 134例患者有生存随访结果，数据显示我中心患者中位OS期48（95％ *CI* 40～56）个月，IWG-PM组患者中位OS期45（95％ *CI* 40～49）个月，两组间差异无统计学意义（*P*＝0.449）（图2A）。

**图2:**
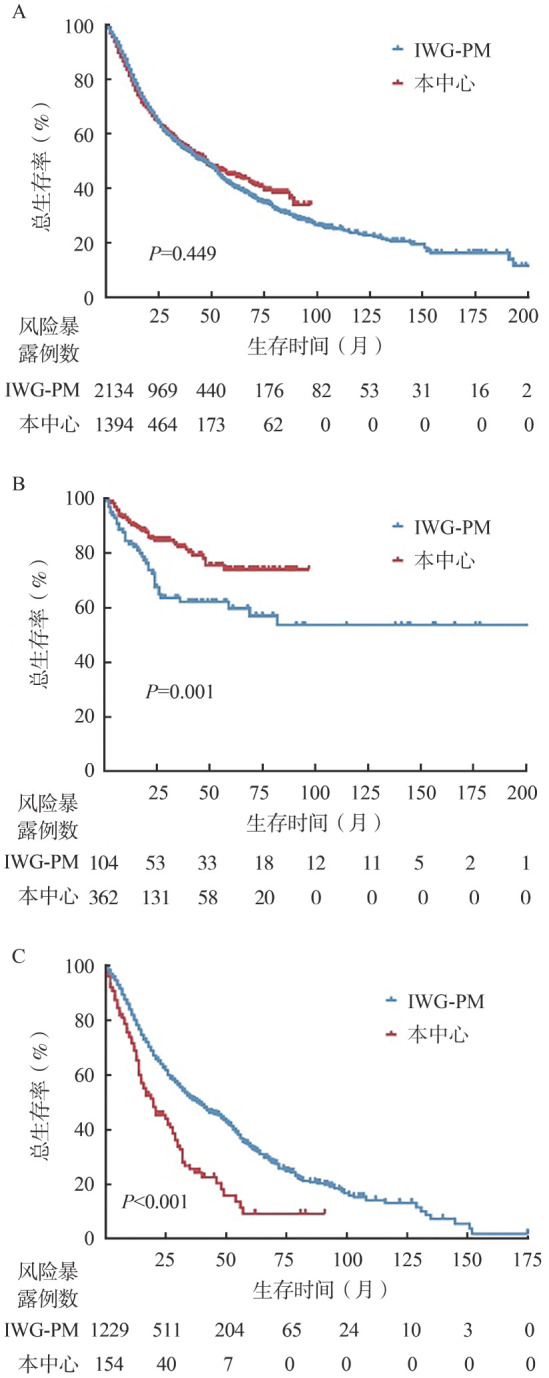
本中心MDS患者与IWG-PM患者生存比较 **A** 总体患者；**B** 年龄≤45岁亚组；**C** 年龄≥70岁亚组 **注** MDS：骨髓增生异常肿瘤；IWG-PM：MDS国际预后工作组队列

二、本中心与IWG-PM队列≤45岁患者临床、实验室特征和生存比较

1. 临床特征：我中心377例患者与110例IWG-PM患者相比更年轻（中位年龄36岁对39岁，*P*＝0.001），性别构成差异无统计学意义（男性59.2％对50.0％，*P*＝0.101），IWG-PM组患者WBC（3.10×10^9^/L对2.93×10^9^/L，*P*＝0.001）、ANC（1.43×10^9^/L对1.08×10^9^/L，*P*＝0.009）和HGB（90.0 g/L对78.0 g/L，*P*<0.001）更高，PLT更高且在0.1的水平上差异有统计学意义（84×10^9^/L对61×10^9^/L，*P*＝0.098），WHO 2016诊断分型两组差异无统计学意义（*P*＝0.205）。

2. 细胞遗传学特征：对于可评估的染色体核型分析，我中心340例患者相较108例IWG-PM组患者正常核型比例更低（46.8％对58.3％，*P*＝0.046），而del（20q）（5.6％对0.9％，*P*＝0.057）和+8（11.8％对5.6％，*P*＝0.070）比例更高，在0.1的水平上差异有统计学意义；del（5q）、−Y、−7/del（7q）和复杂核型比例差异无统计学意义。两组患者IPSS-R细胞遗传学分组和IPSS-R预后分组差异亦无统计学意义（*P*>0.05）（表2）。

3. 分子遗传学特征：二代测序检测结果对比分析示，本中心年轻患者最常发生U2AF1（34.2％）、ASXL1（16.8％）、RUNX1（7.5％）、SETBP1（5.9％）、BCOR（5.9％）突变，相较IWG-PM患者U2AF1突变率显著更高（34.2％对4.5％，*P*<0.001），而SF3B1（5.6％对14.4％，*P*＝0.004）、TET2（4.3％对11.7％，*P*＝0.010）、TP53（3.7％对9.0％，*P*＝0.042）和KMT2D（3.7％对9.9％，*P*＝0.015）突变率更低（图1B）。两组患者IPSS-M预后分组差异有统计学意义（*P*＝0.014）（表2），本中心患者IPSS-M较高危组患者比例更高，但差异无统计学意义（62.3％对52.9％，*P*＝0.106）。

4. 生存分析：对有随访数据的362例本中心患者和104例IWG-PM组患者进行生存比较，我中心移植患者141例（39.0％），均作为删失处理，两组患者中位生存差异有统计学意义（中位OS均未达到，*P*＝0.001）（图2B）。我中心患者1年OS率91.2％，4年OS率75.5％；IWG-PM组患者1年OS率83.3％，4年OS率62.0％（*P*＝0.001）。

三、本中心与IWG-PM队列≥70岁患者临床、实验室特征和生存比较

1. 临床特征：年龄≥70岁的患者中，我中心168例患者相较1 259例IWG-PM患者更年轻（73岁对78岁，*P*<0.001），男性比例更高（72.6％对62.2％，*P*＝0.008），三系血细胞计数更低（*P*<0.001）。WHO 2016诊断分型差异与前述整体差异基本一致，我中心MDS-EB比例更高（47.0％对32.6％，*P*<0.001），MDS-del（5q）比例更低（1.8％对6.4％，*P*＝0.013）。

2. 细胞遗传学特征：本中心与IWG-PM组分别有149例（88.7％）和1 215例（96.5％）患者有可评估的染色体核型，结果显示IWG-PM组患者有更高比例的del（5q）（8.1％对1.4％，*P*＝0.001）和正常核型（60.8％对50.0％，*P*＝0.013），我中心患者有更高比例的del（20q）（7.5％对3.3％，*P*＝0.016）和复杂核型（19.9％对8.9％，*P*<0.001），−Y、+8比例组间差异无统计学意义（表2）。按照IPSS-R预后分组，IWG-PM组较低危组患者比例更高（64.1％对40.3％，*P*<0.001）。

3. 分子遗传学特征：我中心老年患者最常检出的基因突变为ASXL1（25.1％）、DNMT3A（19.8％）、TET2（19.2％）、SF3B1（18.6％）和TP53（16.2％）。相较IWG-PM组，我中心患者有更高比例的NPM1突变（3.0％对0.4％，*P*＝0.004），但SF3B1（18.6％对29.6％，*P*＝0.002）和TET2（19.2％对34.1％，*P*<0.001）基因突变率更低（图1C、表2）。我中心IPSS-M较高危组患者比例更高（69.2％对41.1％，*P*<0.001）。

4. 生存分析：我中心154例患者较IWG-PM组1 229例患者中位OS更短［20（95％ *CI* 13～27）个月对37（95％ *CI* 32～42）个月，*P*<0.001］（图2C）。

## 讨论

本研究首先整体对比中西方患者的临床、实验室特征和生存期，结果显示我中心患者更年轻，这与以往国内外文献报道一致，西方国家MDS患者年龄较我国患者年老10～15岁[4]–[7]。同时本研究显示本中心患者较西方国家患者血细胞减少更严重，有更高比例的MDS-MLD、MDS-EB，而MDS-RS-SLD/MLD和MDS-del（5q）比例较低；在细胞遗传学方面，本中心患者单独出现del（20q）、+8及复杂核型比例较西方国家高，而正常核型、del（5q）、−Y比例较低，这与既往研究结果基本一致[8],[14]–[15]；在分子遗传学异常方面，本中心患者U2AF1、NRAS、NPM1基因突变更常见，而ASXL1、SF3B1、RUNX1等基因突变较西方国家患者少见，基因突变检出率的中西差异与此前我中心和国内外其他单中心研究报道一致[16]–[18]。

在中西方患者年龄、临床和实验室特征有差异的情况下，我们首先考虑年龄差距是否源于我国患者就诊上的偏倚，例如是否存在老年患者因乏力等症状而倾向于在当地医院就诊导致我院患者相对年轻的可能。但同时我中心患者较IWG-PM患者血细胞减少更严重，如果贫血症状重的患者因乏力倾向于在当地就诊，就会使我中心接诊贫血症状相对更轻的患者，这似乎与研究结果不符。同时，我们发现我国其他地区医院报道的MDS患者中位年龄多为55～65岁[18]–[20]，这与我中心患者年龄接近而较西方报道中的MDS患者年轻。另外，既往文献显示，除我国以外，韩国、日本、泰国等其他亚洲国家MDS患者均较西方国家患者年轻[6],[8],[21]–[25]，因此中西方患者年龄差异无法完全用偏倚解释，而更可能是中西方人种差异所致。

其次，由于年轻MDS患者与年老患者疾病表现不同[10],[26]–[28]，中西方患者临床及实验室特征的差异可能与年龄差距相关，于是我们在年轻（≤45岁）和年老（≥70岁）患者中再次比较了中西方患者临床和实验室特征及生存。结果显示缩小年龄差距后的两组患者临床及实验室特征仍然存在差异，我中心患者血细胞减少仍较IWG-PM患者更严重，正常核型比例低而del（20q）比例更高，SF3B1、TET2基因突变率更低而NPM1突变率更高。在年轻患者中，我中心患者染色体+8和U2AF1基因突变更常见，1年、4年生存率均较高，但可能与我中心患者移植等删失患者比例高有关；在老年患者中，我中心患者较西方国家患者有更多复杂核型、更少del（5q），生存期更短。这进一步表明中西方MDS存在无法完全用年龄解释的差异。

西方国家也有关于人种影响患者年龄和临床表现与预后的研究，如非西班牙裔黑种人患者较非西班牙裔白种人和西班牙裔患者生存期更长[29]，黑种人患者较白种人患者更年轻、预后更好[30]等。人种差异的具体原因目前并不明确，可能与遗传背景、生活环境、生活方式、人口年龄构成等因素有关[8],[14]。人种差异可能引起诊断标准、预后工具、新药物临床试验纳入标准以及疗效评估标准普适性不足的问题[31]，因此，我们需要考量国际标准是否适用于中国患者，如我中心既往验证了WHO2022和ICC诊断分型标准可有效区分我国患者群体[32]–[33]，而IPSS-M在我国60岁以上患者中预后效能更高[10]等。

综上，本研究通过大系列比较进一步证实我国与西方国家的MDS患者在临床、实验室特征方面存在人种差异，年龄接近的中西方患者间差异仍然显著。尽管中西方患者总体生存无显著差异，但年轻和老年患者的生存期在中西方之间存在差异，未来仍有必要开展国际标准在中国患者中的验证和调整，以使我国患者得到更精准诊断与治疗。本研究的不足之处是我国患者资料仅来自单中心，仍需多中心患者数据来加以进一步确证。
